# Effects of Vitamin D Plus Calcium Supplements on Pharmacokinetics of Isoflavones in Thai Postmenopausal Women

**DOI:** 10.1155/2011/895471

**Published:** 2011-05-03

**Authors:** Supanimit Teekachunhatean, Paveena Pongnad, Noppamas Rojanasthein, Maleeya Manorot, Chaichan Sangdee

**Affiliations:** ^1^Department of Pharmacology, Faculty of Medicine, Chiang Mai University, Chiang Mai 50200, Thailand; ^2^Center of Thai Traditional and Complementary Medicine, Faculty of Medicine, Chiang Mai University, Chiang Mai 50200, Thailand

## Abstract

The objective of this study was to determine the effects of vitamin D_3_ plus calcium supplements (D_3_-calcium) on pharmacokinetics of isoflavones in Thai postmenopausal women. This study was an open-labeled, randomized three-phase crossover study. Twelve healthy subjects were randomized to receive one of the following regimens: (a) a single dose of isoflavones, (b) a single dose of isoflavones, and D_3_-calcium, or (c) continuous D_3_-calcium for 7 days followed by a single dose of isoflavones on the 8th day. After a washout period, subjects were switched to receive the 2 remaining regimens according to their randomized sequences. Blood samples were collected before dose and at specific time points until 32 hours after isoflavone administration. Plasma was treated with *β*-glucuronidase/sulfatase to hydrolyze glucuronide and sulfate conjugates of daidzein and genistein. Plasma concentrations of daidzein and genistein were determined by high performance liquid chromatography. The estimated pharmacokinetic parameters of isoflavones were time to maximal plasma concentration (T_max_), maximal plasma concentration (C_max_), half-life (t_1/2_) and area under the plasma concentration-time curve (AUC). T_max_ of daidzein and genistein after regimen B was significantly longer than that of regimen A. Other pharmacokinetic parameters of daidzein and genistein obtained following the three regimens were not significantly different.

## 1. Introduction

Menopause is the permanent cessation of menstruation and ovarian function characterized by a lack of estrogen production [1]. Acute estrogen deficiency may cause menopausal symptoms that include a variety of symptoms, such as vasomotor instability (hot flashes and night sweats), insomnia, palpitations, anxiety, urinary symptoms and urethral syndrome, vaginal dryness, dyspareunia, and loss of libido, whereas chronic estrogen deficiency may result in osteoporosis, strokes, and cardiovascular diseases [2]. Estrogen therapy is highly effective for relieving menopausal symptoms and for preventing osteoporosis [3]. Nonetheless, the use of estrogen increases the risk of endometrial cancer [4], whereas this adverse incidence could be avoided by using continuous combined estrogen-progestin replacement therapy [5]. However, recent evidence from the Women's Health Initiative (WHI) clinical trial suggests that estrogen plus progestin initially increases the risk of coronary heart disease, stroke, and venous thromboembolism [6].

The concern over the potential adverse effects of hormonal replacement therapy (HRT) leads many women to look for other alternatives. One modality that has been widely investigated is soy supplement. Soy foods contain isoflavones that are structurally similar to estrogen but have weaker hormonal effects: binding weakly to the estrogen receptor *α* of the uterus, ovaries, and breast, but more strongly to the estrogen receptor *β* that is found in the bone [7]. Soybean protein and foods containing soy are an integral part of many Asian diets and contain significant amounts of isoflavones either as the unconjugated aglycones, such as daidzein and genistein, or as different types of their respective glycoside conjugates, such as daidzin and genistin [7]. Isoflavones have received considerable attention for their potential role in the alleviation of hot flushes [8] as well as reducing the risk of osteoporosis [9, 10] in postmenopausal women.

Generally, adequate supplementation of calcium and vitamin D is recommended as a cornerstone for the prevention and treatment of osteoporosis [11]. A daily intake of at least 1,200 mg of calcium is recommended for all women with osteoporosis [12]. In addition, there is compelling scientific evidence that oral vitamin D reduces the risk of hip and any nonvertebral fractures in ambulatory or institutionalized elderly persons [13]. Nonetheless, oral vitamin D appears to reduce the risk of hip fractures only when calcium supplementation is added [14].

 Thus, it is possible that both peri- and postmenopausal women might consider using the combination of vitamin D, calcium, and soy isoflavones for the prevention and treatment of postmenopausal osteoporosis instead of HRT. Nonetheless, the pharmacokinetic interactions between these agents have not been reported as of yet. Thus, the purpose of this study was to determine the effects of vitamin D plus calcium supplements on pharmacokinetics of isoflavones in Thai postmenopausal women.

## 2. Materials and Methods

### 2.1. Study Design

 This study was a randomized, open-labeled, crossover-three phase study, with washout periods of at least 2 weeks. The study was approved by the Human Research Ethics Committee of the Faculty of Medicine, Chiang Mai University, and complied with the Helsinki Declaration.

### 2.2. Subjects

 A total of 12 postmenopausal women aging ≥45 years exhibiting serum follicle-stimulating hormone concentration >20 IU/L were enrolled in this study. All had to be in good general health on the basis of medical history and a thorough physical examination. Routine blood examinations including complete blood count, blood urea nitrogen, serum creatinine, and liver function test were carried out to exclude subjects with abnormal hematological diseases or impaired kidney and liver function. Blood concentrations of calcium and phosphate had to be within normal ranges of 8.5–10.5 and 2.5–5.0 mg/dL, respectively. The body mass index of all subjects had to be within 18–25 kg/m^2^. The subjects were advised to maintain their usual diet and to avoid consuming high quantities of vitamin D or a high calcium diet as well as to avoid soy food throughout the study. Supplements of vitamin D, calcium, and isoflavones were not allowed throughout the study. Exclusion criteria were subjects with known premenopausal status (<12 months since the last spontaneous menstrual bleeding), history of chronic renal, liver, pulmonary, or cardiovascular diseases, recent cigarette smoking, substance abuse or addiction, use of antibiotics within the previous 6 weeks, consumption of >2 alcoholic drinks/day, regular use (>1 dose/week) of over-the-counter or prescription medications, as well as a history of malignancy, hyperparathyroidism, or breast disease.

### 2.3. Isoflavones and Vitamin D_3_ Plus Calcium (D_3_-Calcium) Supplements

 The isoflavone preparation used in this study was the commercial soy isoflavone capsule, Flava soy (1 capsule contains not less than 25 mg isoflavones, manufactured by Thai Herbal Products Co., Ltd, Thailand). D_3_-calcium supplement was the commercial Caltrate 600 + D (1 tablet contains vitamin D_3_ 200 IU plus calcium 600 mg, manufactured by Wyeth-Ayerst Co., Ltd, Thailand).

### 2.4. Dosage and Drug Administration

 Subjects were admitted to the Clinical Pharmacology Unit, Faculty of Medicine, Chiang Mai University at 6 : 30 AM after an overnight fast of at least 8 h. Subjects were randomized to receive (a) a single dose of 2 capsules of Flava soy (single ISO), (b) a single dose of 2 capsules of Flava soy and 1 tablet of Caltrate 600 + D (“single ISO + D_3_-calcium”), or (c) 1 tablet of Caltrate 600 + D twice a day after breakfast and dinner for 7 days, followed by 2 capsules of Flava soy on the 8th day (continuous D_3_-calcium/single ISO). Subjects were asked to remain upright and asked to fast for 2 hours (h) after isoflavone administration. Water and lunch were served at 2 h and 6 h after dosing, respectively. Blood samples were collected at specific time points (see below). After blood sample collection at 12 h afterdose, subjects were discharged and were asked to come back again on the next day for blood sample collections at 24 h and 32 h afterdose. After a washout period of at least 2 weeks, subjects were switched to receive the two remaining regimens according to their randomized sequences. Subjects were required to refrain from drinking caffeine and alcohol containing beverages in order to standardize experimental conditions.

### 2.5. Blood Sample Collection

 Venous blood samples (10 mL/each) for the determination of soy isoflavones were collected beforedose and then at 1, 2, 4, 6, 8, 10, 12, 24, 32 h after isoflavone administration. Samples were obtained from the forearm by venipuncture through an indwelling intravenous catheter (BD Insyte) and collected in heparinized vacutainer (BD Insyte). The blood collecting tubes were centrifuged at 2,500 rpm for 10 min, and the plasma was separated and frozen at −80°C until analysis.

### 2.6. Determination of Isoflavone Concentrations

#### 2.6.1. High-Performance Liquid Chromatography (HPLC) Condition

 The assay of isoflavone content was modified from the HPLC method and conditions previously described by Thomas et al. [15]. Chromatographic separation was performed on 5 *μ*m C18, 100×4.6 i.d. analytical and guard columns. The chromatography condition consisted of 2 mobile phases. Mobile phase A was 250 : 105 : 105 (v/v/v) and Mobile phase B was 250 : 200 : 250 (v/v/v) of 40 mM ammonium acetate/acetonitrile/methanol, respectively. Twenty *μ*L of perchloric acid and 250 *μ*L of 1.44 mM sodium dodecyl sulfate were added into both mobile phases. The HPLC system was run by 100% mobile phase A for 2.50 min and followed by 100% mobile phase B for 11.50 min. The flow rate was maintained at 1 mL/min, the column was maintained at 25°C, and the analytes were detected by UV absorption at 259 nm.

#### 2.6.2. Sample Preparation

 Initially, the mixture of *β*-glucuronidase/sulfatase enzyme was made up freshly by adding 500 *μ*L of *β*-glucuronidase/sulfatase from *Helix pomatia* (Sigma G-0876) into 10 mL of 0.1 M sodium acetate containing 0.1 g ascorbic acid and 0.01 g ethylenediaminetetraacetic acid (EDTA). Aliquot of 125 *μ*L of plasma was transferred to a 1.5 mL plastic vial and treated with 0.25 mL of a mixture of *β*-glucuronidase/sulfatase to hydrolyze glucuronide and sulfate conjugates of daidzein and genistein. The tubes were capped and heated overnight in water bath (37°C, 15–18 h), then removed from the bath, and allowed to cool at room temperature.

#### 2.6.3. Determination of Isoflavone Concentrations in Plasma

**Figure 1:**
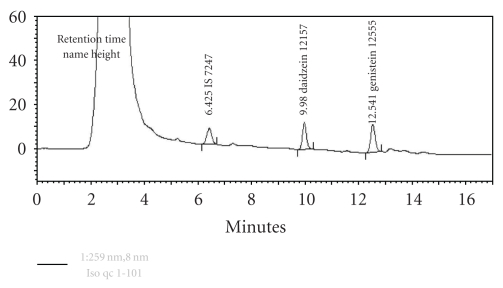
Chromatogram of plasma sample containing 2,400 ng/mL of daidzein (retention time, k = 9.980 min) and genistein (k = 12.541 minutes) and 4,000 ng/mL of fluorescein (internal standard, k = 6.425 minutes).

The assay was modified from protein precipitation procedure. Briefly, after enzymatic hydrolysis, plasma samples were spiked with 10 *μ*L of internal standard (IS, 50,000 ng/mL fluorescein in 80% methanol) and then deproteinated by mixing plasma samples with 500 *μ*L acetonitrile, vortex mixing for 30 sec and centrifuged at 14,000 rpm for 10 min, respectively. An aliquot of the supernatant was removed and evaporated for 2 h at 60°C. The residue was dissolved in 50 *μ*L of the mobile phase, and 5 *μ*L of the sample was injected onto the HPLC system. Chromatogram of plasma containing daidzein, genistein, and IS is presented in Figure 1. The isoflavone contents of samples were determined by using a calibration curve of peak height ratios of isoflavones (daidzein and genistein) and IS versus respective isoflavone concentrations (37.5, 75, 150, 300, 600, 1200, and 2400 ng/mL) with the use of linear regression. The linear regression analysis of peak height ratios of isoflavones versus isoflavone concentrations consistently yielded coefficients of determinant (*r* ^2^) of 0.997 or better.

Intraday and interday precisions were determined using 3 quality control samples of daidzein and genistein in plasma (112.5, 1100, and 2200 ng/mL). For determination of daidzein concentrations in plasma, the mean percentages of coefficient of variation (%CV) of intraday and interday precision were 4.10% and 6.86%, respectively, whereas the mean deviation of intraday and interday assay were 4.27% and −0.41%, respectively. The mean recovery of daidzein from the determination procedure was 89.63%. For determination of genistein concentrations in plasma, the mean %CV of intraday and interday precision was 4.36% and 5.48%, respectively, whereas the mean deviation of intraday and interday assay was 10.37% and 7.37%, respectively. The mean recovery of genistein from the determination procedure was 88.58%.

### 2.7. Data Analysis and Statistical Methods

#### 2.7.1. Pharmacokinetic Parameters

Time to reach peak concentration (T_max_, h) and maximal plasma concentration (C_max_, ng/mL) was obtained directly by visual inspection of each subject's plasma concentration-time profile. The slope of the terminal log-linear portion of the concentration-time profile was determined by least-squares regression analysis and was used as the elimination rate constant (*Ke*). The elimination half-life (t_1/2_) was calculated as 0.693/*Ke*. The AUC from time zero to the last quantifiable point (AUC_0-32_) was calculated using the trapezoidal rule. Extrapolated AUC from the last quantifiable time to infinity (AUC_32-∞_) was determined as Ct/*Ke*. Total AUC_0-∞_ was the sum of AUC_0-32 _+ AUC_32-∞_. The calculation was performed using the TopFit software version 2.0 for PC.

#### 2.7.2. Statistical Analysis

The pharmacokinetic parameters were presented as mean ± standard deviation (SD). The mean values of pharmacokinetic parameters obtained from regimen A (single ISO) were compared to those of the two remaining regimens using Wilcoxon signed-rank test.

## 3. Results

The demographic characteristics of 12 subjects enrolled in the study are shown in Table 1. All completed study protocol; however data from one subject was excluded from analysis since isoflavones were detected in her serum at baseline of “single ISO + D_3_-calcium” phase. Every subject was healthy on the basis of medical history, as well as physical and biochemical investigations.

**Figure 2:**
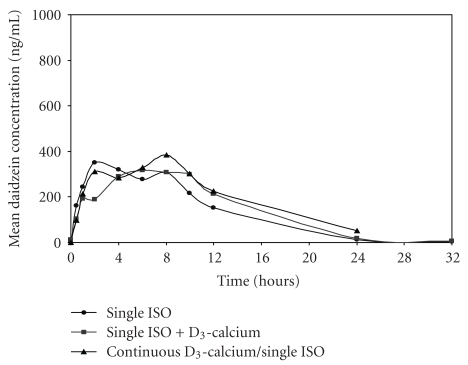
Mean plasma daidzein concentration-time curves after receiving three different regimens obtained from subjects who completed the study without protocol deviation (*n* = 11). Each point on the curves represents mean plasma concentration at the corresponding time point. *Single ISO*: a single dose of Flava soy; *Single ISO + D_3_-calcium*: a single dose of Flava soy and Caltrate 600 + D; *Continuous D_3_-calcium/single ISO*: 1 tablet of Caltrate 600 + D twice a day for 7 days, followed by a single dose of Flava soy on the 8th day.

**Figure 3:**
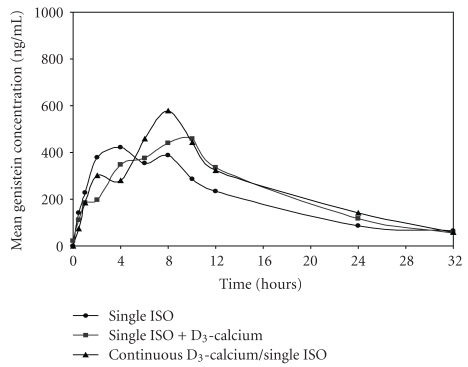
Mean plasma genistein concentration-time curves after receiving three different regimens obtained from subjects who completed the study without protocol deviation (*n* = 11). Each point on the curves represents mean plasma concentration at the corresponding time point. *Single ISO*: a single dose of Flava soy; *Single ISO + D_3_-calcium*: a single dose Flava soy and Caltrate 600 + D; *Continuous D_3_-calcium/single ISO*: 1 tablet of Caltrate 600 + D twice a day for 7 days, followed by a single dose of Flava soy on the 8th day.

 The mean plasma concentration-time curves after taking three different regimens of soy extract are shown in Figures 2 and 3. The plasma concentration-time profiles of daidzein and genistein were typically biphasic regardless of soy extract regimens taken. The first and second peak concentrations of both aglycones were generally attained at 2–4 h and 6–8 h, respectively. The mean values of pharmacokinetic parameters of daidzein and genistein after receiving three different regimens are shown in Tables 2 and 3. 

Of all pharmacokinetic parameters, only T_max_ of daidzein and genistein after “single ISO + D_3_-calcium” was significantly longer than “single ISO” (Tables 2 and 3). Other pharmacokinetic parameters (C_max_, AUC_0-32_, AUC_0-∞_, and t_1/2_) of daidzein and genistein analyzed after the three different regimens were not significantly different.

## 4. Discussion

 This is the first study to investigate the interaction between vitamin D and calcium with soy isoflavones. Since the study design of this study was similar to that of the bioequivalence testing, 12 subjects were enrolled in this study according to the minimum number of subjects stipulated by the Canadian and European guidelines for bioequivalence testing. 

 Theoretically, the isoflavones in nonfermented soy food appear mostly as the glycoside conjugates, whereas the aglycones (e.g., daidzein, genistein, etc.) dominate in fermented soy products [16]. Since the amounts of daidzein and genistein detected in the Flava Soy capsule were negligible (data not shown), it was postulated that the predominant forms of isoflavones existing in the preparation were glycoside conjugates. However, because human intestinal or gut microfloral glucosidases seemingly cleave these moieties before these isoflavones can be absorbed, most of the absorbed isoflavones are aglycones [7]. The absorbed aglycones are then largely converted to their *β*-glucuronides by enzymes in the gut wall, which may be a major site of glucuronidation, and also in the liver after they reach systemic circulation. In fact, isoflavones are predominantly conjugated with glucuronic acid and, to a lesser extent, with sulfate [17]. The purpose of using a mixture of *β*-glucuronidase/sulfatase in the quantification of plasma isoflavone concentrations in this study was to hydrolyze glucuronide and sulfate conjugates to aglycones. Thus, plasma concentrations of daidzein and genistein were determined rather than their glucuronide and sulfate conjugates.

 In this study, the biphasic pattern observed from the concentration-time curve of both aglycones indicates an enterohepatic recirculation as already suggested by several authors [18–20]. The first peak corresponds to absorption occurring readily in the small intestine [18, 19, 21] where the glycoside forms of isoflavones are hydrolyzed by glucosidases to the aglycone forms [22]. The second peak possibly results from the entero-hepatic recirculation of the glucuronide and sulfate conjugates of isoflavones excreted in the bile [19]. In this study, the second peak concentrations of both isoflavones were attained approximately at 2 h after lunch in most subjects; this finding supports the existence of entero-hepatic recirculation. Additionally, the long transit time of unabsorbed glycosides reaching the colon and subsequently cleaved by microfloral glucosidases may also help to explain the second surge of plasma daidzein and genistein concentrations [23].

 The mean T_max_ of daidzein (5.50 + 2.11 h) and genistein (4.58 + 2.11 h) was quite shorter than those of approximately 8-9 h after ingestion of isoflavone conjugates reported in Caucasian subjects [18, 20]. This discrepancy might be the result from such factors as race, uptake rates, and rapidity of hydrolysis of glycosides by gut bacteria or gut wall enzymes. However, the difference in the rate of cleavage of these glycosides by gut wall enzymes and bacteria among the different populations seems to be a more plausible explanation since the T_max_ of these aglycones of the present study was more comparable to another study done in the Asian population [19, 24].

 “Single ISO + D_3_-calcium” significantly delayed T_max_ of both daidzein and genistein compared to “single ISO.” The reason behind the delay in T_max_ by D_3_-calcium is unknown. Since aglycones are absorbed by passive diffusion [25], and there is no evidence for facilitated or active transport of isoflavones, it seems unlikely that D_3_-calcium exerts this interference via the involvement of a carrier or saturation process. However, this delayed rate of absorption might be the result from at least 3 possibilities: firstly, since it has been shown that the rate-limiting step for absorption is initial hydrolysis of sugar moiety [26], therefore, either vitamin D or calcium (or both) possibly and transiently inhibits glucosidase activity leading to the delayed cleavage of these glycoside conjugates into aglycones and thus increases in their T_max_. Secondly, calcium carbonate in Caltrate 600 + D may cause delayed gastric emptying time leading to the delayed T_max_ [27]. Thirdly, some inactive ingredients in Caltrate 600 + D might alter the bioavailability of aglycones because it has been shown that fiber and carbohydrates are associated with differences in the absorption of aglycones [28]. Among these possibilities, the latter appears unlikely because the amount of inactive ingredients would be too little to cause such a significant delay.

 Although “single ISO + D_3_-calcium” significantly delayed the T_max_ of both isoflavones, single dose and multiple doses of D_3_-calcium did not significantly affect other pharmacokinetic characteristics of daidzein and genistein since C_max_, AUC, and t_1/2_ of these isoflavones were not significantly different among the three regimens. Therefore, D_3_-calcium only delayed the absorption of isoflavones without significant effects on the extent of isoflavones absorbed or on their metabolism. However, we postulate that these drug interactions cause a negligible effect on isoflavones' therapeutic outcomes because reduced absorption rate is only important if a rapid pharmacological effect is sought which depends on attaining a high peak concentration (e.g., with antibiotics and analgesics) [29]. Therefore, coadministration of D_3_-calcium and isoflavones seems to be the simple method to enhance drug compliance, especially in the elderly patients taking several medications, and this advantage might outweigh the minor effect of D_3_-calcium on pharmacokinetics of isoflavones. Indeed, the D_3_-calcium-isoflavones should be encouraged for the treatment of some indications (such as postmenopausal osteoporosis) and might become an appropriate alternative food supplementation to HRT in postmenopausal women in the near future. Nevertheless, the clinical studies in this aspect should be further investigated. 

 The major limitation of this study was the lack of quantification of isoflavone conjugates (daidzin and genistin) in the soy preparation used; the variations in amount per capsule may contribute to the remarkably high variations in C_max_ and AUC of aglycones among the different regimens, even in the same subjects. In addition, lack of verification of dissolution of the soy formulation was another limitation, yielding the unanswered question of whether delayed absorption of aglycones was due to the different dissolution rate or drug interaction. The small sample size also contributes to a marked variation encountered in this study. Future studies should be designed to overcome these limitations by determination of isoflavone contents existing in the study preparation, as well as their dissolution profiles, and by using a larger sample size. Besides, although the washout period of at least 2 weeks assigned in this study might be sufficiently long comparing to plasma t_1/2_ of vitamin D (191–25 h) and isoflavones, it seems to be rather short comparing to tissue t_1/2_ of vitamin D stored in fat depots. This might provide potential carryover effects of the vitamin D intervention on the subsequent study phases. Finally, since serum D_3_ production is influenced by an exposure to sunlight, lack of assessment and control of sunlight exposure in the individual subject should be considered as another limitation.

## 5. Conclusion

Simultaneous administration of D_3_-calcium delayed T_max_ of daidzein and genistein without significant alteration in C_max_, AUC_0-32_, AUC_0-∞_, and t_1/2_ of both aglycones.

## Figures and Tables

**Table 1:** The demographic characteristics of subjects enrolled in the study (*n* = 12).

Subject	Age	Weight	Height	BMI	FSH
No.	(y)	(kg)	(m)	(kg/m^2^)	(IU/L)
1	52	57.00	1.60	22.27	49.20
2	53	52.50	1.53	22.43	48.50
3	54	54.00	1.51	23.68	33.50
4	54	60.00	1.56	24.65	49.40
5	56	48.50	1.45	23.07	38.60
6	56	42.00	1.44	20.25	68.50
7	49	58.50	1.66	21.23	128.20
8	55	58.00	1.53	24.78	68.80
9*	57	48.50	1.50	21.56	101.00
10	62	60.50	1.60	23.63	53.33
11	59	49.00	1.51	21.50	40.79
12	62	48.00	1.42	23.81	63.49

Mean	55.75	53.04	1.53	22.74	61.94
SD	3.70	5.63	0.07	1.37	26.43

*Data from subject no. 9 was ruled out because isoflavone concentration was detected in plasma sample at baseline of “single ISO + D_3_-calcium” phase.

**Table 2:** Pharmacokinetic parameters of daidzein after receiving three different regimens.

Pharmacokinetic parameters^†^	Treatment regimens		
	Single ISO	Single ISO +D_3_-calcium^‡^	Continuous D_3_-calcium/single ISO
T_max_ (h)	5.50	8.00*	6.08
	(2.11)	(1.79)	(2.50)
C_max_ (ng/mL)	506.09	396.32	488.05
	(299.62)	(115.37)	(165.90)
t_1/2_ (h)	6.80	6.34	6.26
	(2.98)	(5.29)	(3.73)
AUC_0-32_ (ng·h/mL)	3474.05	2968.71	4620.55
	(931.53)	(627.60)	(2148.09)
AUC_0-∞ _(ng·h/mL)	4905.23	4938.40	5961.22
	(1150.99)	(1611.00)	(2546.45)

Data represents mean (SD). ^†^The parameters were derived from analysis of the individual concentration-time curve. ^‡^Data from one subject was ruled out because isoflavone concentration was detected in plasma sample at baseline. *Single ISO*: a single dose of Flava soy; *Single ISO + D_3_-calcium*: a single dose of Flava soy and Caltrate 600 + D; *Continuous D_3_-calcium/single ISO*: 1 tablet of Caltrate 600 + D twice a day for 7 days, followed by a single dose of Flava soy on the 8th day. *P < .05 versus Single ISO.

**Table 3:** Pharmacokinetic parameters of genistein after receiving three different regimens.

Pharmacokinetic parameters^†^	Treatment regimens		
	Single ISO	Single ISO +D_3_-calcium^‡^	Continuous D_3_-calcium/single ISO
T_max_ (h)	4.58	7.64*	6.08
	(2.11)	(2.16)	(2.78)
C_max_ (ng/mL)	593.33	578.99	688.56
	(303.31)	(240.98)	(285.42)
t_1/2_ (h)	8.31	7.44	8.86
	(4.90)	(2.27)	(4.09)
AUC_0-32_ (ng·h/mL)	5804.19	6574.74	7758.56
	(2643.87)	(2625.30)	(5068.69)
AUC_0-∞ _ (ng·h/mL)	6787.62	7434.65	9091.58
	(2793.66)	(2748.42)	(5394.67)

Data represents mean (SD). ^†^The parameters were derived from analysis of the individual concentration-time curve. ^‡^Data from one subject was ruled out because isoflavone concentration was detected in plasma sample at baseline. *Single ISO*: a single dose of Flava soy; *Single ISO + D_3_-calcium*: a single dose of Flava soy and Caltrate 600 + D; *Continuous D_3_-calcium/single ISO*: 1 tablet of Caltrate 600 + D twice a day for 7 days, followed by a single dose of Flava soy on the 8th day. *P < .05 versus Single ISO.
